# Early Impairment of Synaptic and Intrinsic Excitability in Mice Expressing ALS/Dementia-Linked Mutant UBQLN2

**DOI:** 10.3389/fncel.2016.00216

**Published:** 2016-09-20

**Authors:** Daniel Radzicki, Erdong Liu, Han-Xiang Deng, Teepu Siddique, Marco Martina

**Affiliations:** ^1^Department of Physiology, Northwestern University Feinberg School of MedicineChicago, IL, USA; ^2^Department of Neurology, Northwestern University Feinberg School of MedicineChicago, IL, USA

**Keywords:** ALS/dementia, pyramidal cell, CA1, glutamate

## Abstract

Frontotemporal dementia (FTD) and amyotrophic lateral sclerosis (ALS) are believed to represent the different outcomes of a common pathogenic mechanism. However, while researchers have intensely studied the involvement of motor neurons in the ALS/FTD syndrome, very little is known about the function of hippocampal neurons, although this area is critical for memory and other cognitive functions. We investigated the electrophysiological properties of CA1 pyramidal cells in slices from 1 month-old UBQLN2^P497H^ mice, a recently generated model of ALS/FTD that shows heavy depositions of ubiquilin2-positive aggregates in this brain region. We found that, compared to wild-type mice, cells from UBQLN2^P497H^ mice were hypo-excitable. The amplitude of the glutamatergic currents elicited by afferent fiber stimulation was reduced by ~50%, but no change was detected in paired-pulse plasticity. The maximum firing frequency in response to depolarizing current injection was reduced by ~30%; the fast afterhyperpolarization in response to a range of depolarizations was reduced by almost 10 mV; the maximum slow afterhyperpolarization (sAHP) was also significantly decreased, likely in consequence of the decreased number of spikes. Finally, the action potential (AP) upstroke was blunted and the threshold depolarized compared to controls. Thus, synaptic and intrinsic excitability are both impaired in CA1 pyramidal cells of UBQLN2^P497H^ mice, likely constituting a cellular mechanism for the cognitive impairments. Because these alterations are detectable before the establishment of overt pathology, we hypothesize that they may affect the further course of the disease.

## Introduction

Amyotrophic lateral sclerosis (ALS) is a progressive neurodegenerative disease caused by selective degeneration of motor neurons in the brain and spinal cord. As motor neurons control voluntary movement and respiration, progression of the disease leads to progressive paralysis and eventually respiratory failure and death. ALS has traditionally been classified into two major categories: sporadic and familial. In general, familial ALS has an earlier onset and a slower progression, while sporadic ALS usually appears around 60 years of age (Swinnen and Robberecht, 2014). Over time it has been recognized that cognitive impairment is frequent in ALS and that frontotemporal dementia (FTD) and ALS may represent different outcomes of a common pathogenic mechanism, although the reason why some neurons, typically large spinal and cortical motor neurons, are particularly susceptible remains unclear.

It was previously reported that mutant UBQLN2^P497H^ mice, which reproduce a mutation found in human ALS/FTD patients, are cognitively impaired (Gorrie et al., 2014). This impairment appears linked to proteasome impairment leading to the formation of aggregates in neuronal cell bodies and dendrites, and most markedly in dendritic spines. In UBQLN2^P497H^ transgenic mice dendritic spines of cortical neurons are dramatically enlarged, having sizes up to 5 times the normal (Gorrie et al., 2014). Furthermore, tissue staining with ubiquilin2 antibody reveals the presence of numerous ubiquilin2-positive aggregates in all the main hippocampal subfields (CA1, CA3 and dentate gyrus), and hippocampal LTP is hindered in brain slices obtained from 3 month old UBQLN2^P497H^ mice. Because LTP is the consequence of precisely timed synaptic (excitatory synaptic currents) and intrinsic action potential (AP) activity (Paulsen and Sejnowski, 2000) changes in any of these parameters may underlie the observed impairment. Additionally, altered electrical excitability may affect numerous other functional properties including gene expression (Cohen and Greenberg, 2008). Dysfunctions of neuronal excitability are a constant finding in neurological disease, although the polarity of the changes may vary in different cell types or in different stages of the disease progression. For example, in Alzheimer’s disease, AB oligomers impair synaptic plasticity (Shankar et al., 2008), but intrinsic excitability appears to increase, at least in the early stages (Hall et al., 2015). In Parkinson’s disease, on the other hand, direct and indirect pathway striatal spiny neurons show alterations of intrinsic excitability of opposite polarity, while synaptic connectivity is only affected in indirect pathway neurons (Fieblinger et al., 2014). Thus, although a link between brain dysfunction and neuronal excitability is always present, the type (synaptic vs. intrinsic) and polarity of the alterations are often unpredictable.

The peculiar spinopathy that characterizes UBQLN2^P497H^ mice strongly suggests that the synaptic responses are affected, yet the presence of either primary or homeostatic changes in intrinsic excitability is also to be considered. Presently, however, nothing is known concerning the electrophysiological properties of hippocampal neurons in these mice at preclinical disease stage. Here we investigated the electrophysiological properties of CA1 pyramidal neurons of 1 month old UBQLN2^P497H^ mice. This is a particularly important time-point because at this age overt pathology is not yet detectable. We found that, at this age-point, glutamatergic signaling is severely impaired in these neurons due to a large reduction in the size of the AMPA-mediated current. Additionally, intrinsic excitability is also widely impaired. The presence of altered membrane excitability at this early stage of the disease may therefore suggest that such change might have a causal role in the progression of the disease.

## Materials and Methods

Hippocampal brain slices: 29- to 35-day-old WT and transgenic UBQLN2^P497H^ mice were anesthetized with isoflurane and killed by decapitation. In these animals the pathology is 100% penetrant.

All experiments followed protocols approved by the Northwestern University Center for Comparative Medicine, an Association for Assessment and Accreditation of Laboratory Animal Care accredited facility, and followed guidelines issued by the National Institutes of Health and the Society for Neuroscience.

The brains were quickly removed from the skull in ice-cold low Ca^2+^, high Mg^2+^ artificial cerebrospinal fluid (ACSF), containing (in mM): 87 NaCl, 25 NaHCO_3_, 2.5 KCl, 1.25 NaH_2_PO_4_, 0.5 CaCl_2_, 7 MgCl_2_, 75 sucrose and 25 glucose, bubbled with 95% O_2_ and 5% CO_2_ (pH 7.4). Transverse hippocampal slices of 300 μm thickness were cut using a vibroslicer (Leica VT1200) and then stored in a solution containing (in mM): 87 NaCl, 25 NaHCO_3_, 2.5 KCl, 1.25 NaH_2_PO_4_, 0.5 CaCl_2_, 7 MgCl_2_, 75 sucrose and 25 glucose, bubbled with 95% O_2_ and 5% CO_2_ (pH 7.4) for 15 min at 35°C and subsequently at room temperature. For the recordings, slices were transferred to a recording chamber bathed in physiological ACSF (in mM: 125 NaCl, 25 NaHCO_3_, 2.5 KCl, 1.25 NaH_2_PO_3_, 25 glucose, 2.0 CaCl_2_ and 1 MgCl_2_, equilibrated with 95% O_2_ and 5% CO_2_) at 30–32°C. Slices were visualized using an upright microscope (Scientifica) using an Olympus 60× water-immersion objective and infrared illumination. Pyramidal cells were identified on the basis of shape, location and electrophysiological properties.

Pipettes were pulled from borosilicate glass using a horizontal puller (P97, Sutter, Novato, CA, USA). For the study of synaptic currents pipettes were filled with a CsCl based internal solution consisting of (in mM): CsCl 138, NaCl 2, MgCl_2_ 2, EGTA 10, HEPES 10, Na_2_ATP 2, NaGTP 0.03, pH 7.3 with CsOH; by blocking voltage gated and leakage potassium currents this solution allows better clamp condition. Furthermore, for these recordings the bath solution contained picrotoxin (0.1 mM) to block GABAergic currents. For the study of intrinsic properties and excitability, no synaptic blockers were added to the extracellular solution and pipettes were filled with an internal solution containing (in mM): KGluconate 140, NaCl 8, MgCl_2_ 2, EGTA 0.1, HEPES 10, Na_2_ATP 2, NaGTP 0.03, pH 7.4 with KOH. Tip resistances in working solutions ranged from 3 to 8 MΩ yielding series resistances of 10–30 MΩ that were compensated by 75–90%. Recordings were performed using an Axopatch 200B amplifier. The reported membrane potential values are reported without correcting for liquid junction potentials.

Membrane potential was measured in current clamp (without any current injection) right after breaking into whole-cell configuration. Input resistance was calculated by dividing the time constant of the peak voltage response onset, obtained using a 1 s-long current injection (−300 pA) from a membrane potential of −70 mV, by the cell capacitance (obtained in voltage clamp by using the capacitance correction circuit of the amplifier). Synaptic stimulation was performed using a concentric bipolar electrode (FHC) positioned in the stratum radiatum at a distance of 125–225 μm from the soma of each patched neuron. This stimulation electrode was connected to a WPI Stimulus Isolator (A360) current stimulator that was controlled using the digital output in PClamp8. The intensity of the stimulus varied between 50 and 800 μA and was increased in 50 or 100 μA steps. For these recordings cells were voltage-clamped at −70 mV and the bath solution contained 100 μM picrotoxin (Abcam).

### Statistical Analysis

Data in the text are reported as mean ± SEM. The error bars in the figures also represent SEM. Statistical significance was assessed using the non-parametric Wilcoxon-Mann-Whitney test at *p* < 0.05 (indicated in Figures by *).

## Results

**Figure 1:**
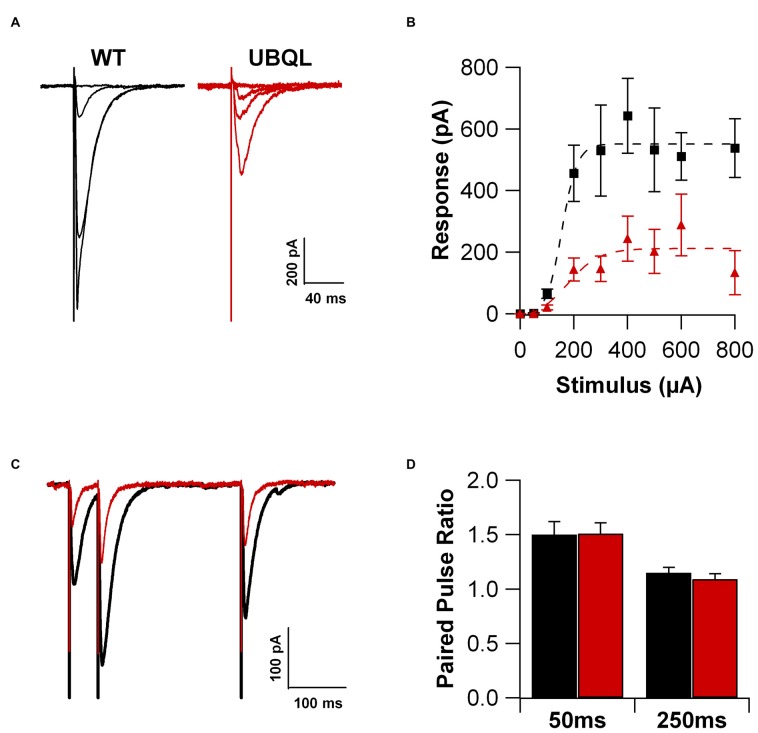
**Glutamatergic current is reduced in UBQLN2^P497H^ mice. (A)** Voltage clamp recordings (Vh = −70 mV) of hippocampal pyramidal cells in slice from a control (black traces) and an amyotrophic lateral sclerosis (ALS; red traces) mouse. **(B)** Summary of the synaptic currents recorded at −70 mV in 10 control and 16 UBQLN2^P497H^ cells in response to extracellular stimulations of increasing magnitude. **(C,D)** No difference was detected in paired pulse ratio between cells from WT and UBQLN2^P497H^ mice (Vh = −70 mV; 9 WT and 12 UBQLN2^P497H^ cells).

We previously found that LTP is impaired in hippocampal slices from 3 month old UBQLN2^P497H^ mice (Gorrie et al., 2014), an age when cellular pathology is clearly detectable. Here, we performed patch clamp recordings from CA1 hippocampal pyramidal neurons in acute slices obtained from 29 to 35 day-old UBQLN2^P497H^ mice, an age in which overt pathology is not yet detectable. As a first step, we investigated the strength of the excitatory input to the pyramidal cells. We hypothesized that the glutamatergic current is smaller in CA1 cells from transgenic mice. To this end, cells were voltage clamped at −70 mV and a postsynaptic current response was elicited by extracellular electrical stimulation of the afferent fibers (200 μs; 50–800 μA, in the stratum radiatum). The maximum current amplitude was 642.8 ± 121.6 pA in cells from control mice, while it was only 243.7 ± 72.9 pA in cells from UBQLN2^P497H^ mice (Figures 1A,B; 10 and 16 cells, respectively; *p* = 0.01 *p* = 0.002). The reduced postsynaptic current response may depend on presynaptic impairment, postsynaptic factors or a combination of the two. In order to test possible alterations in presynaptic release, we compared the paired-pulse ratio (PPR) of these synapses in control and UBQLN2^P497H^ animals. We examined the PPR at 50 and 250 ms time intervals. As previously reported (Chapman et al., 1995), in control animals the synaptic response was strongly facilitated at 50 ms intervals; the magnitude of the facilitation quickly decreased at longer time intervals and it was completely abolished at 500 ms intervals. Interestingly, no difference in PPR was observed between the control and UBQLN2^P497H^ animals at any of the time intervals investigated (9 and 12 cells, respectively, Figures 1C,D) thus suggesting that presynaptic mechanisms are unlikely to play an important role in the reduced glutamatergic current response. Thus, CA1 pyramidal neurons of 1-month old UBQLN2^P497H^ mice display an overall decreased synaptic excitation that is the result of smaller macroscopic currents, while no change was detected in presynaptic release as determined using PPR.

**Figure 2:**
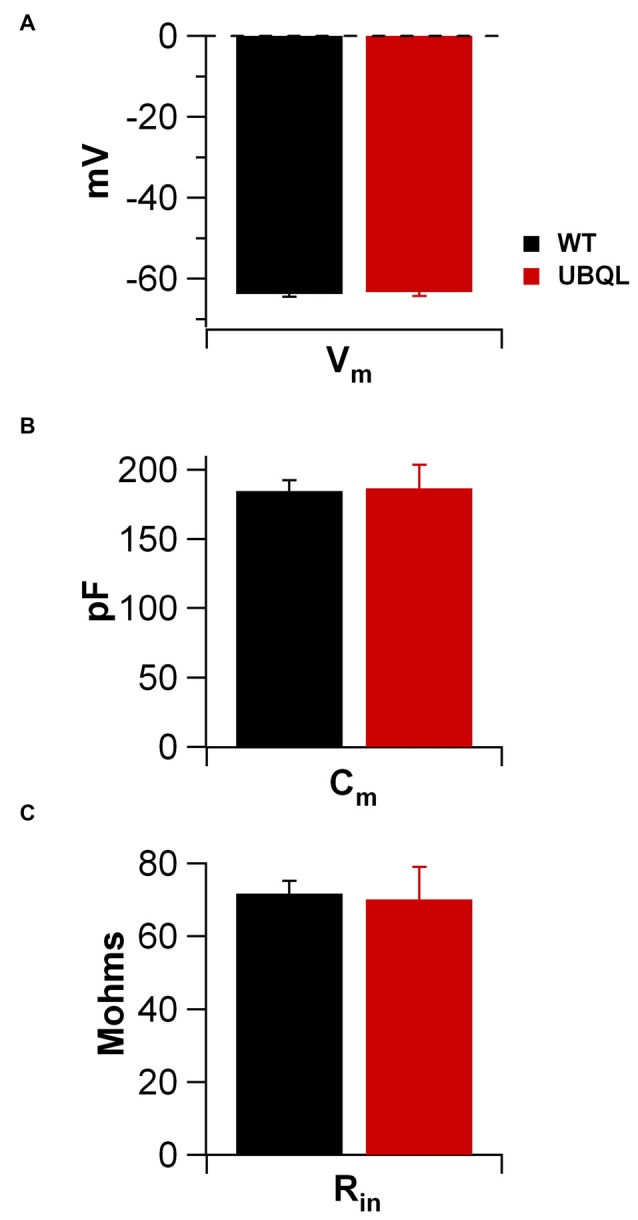
**Membrane properties of CA1 pyramidal cells of control and UBQLN2^P497H^ mice.** No differences were detectable between the basic membrane properties of UBQLN2^P497H^ and WT pyramidal neurons. Bar charts show the summary data for resting membrane potential (**A**; measured upon breaking into the cell; 12 WT and 11 UBQLN2^P497H cells^), membrane capacitance (**B**, 12 and 13 cells, respectively) and input resistance (**C**, 12 and 13 cells, respectively). Internal solution for all these measurements was K-gluconate based.

**Figure 3:**
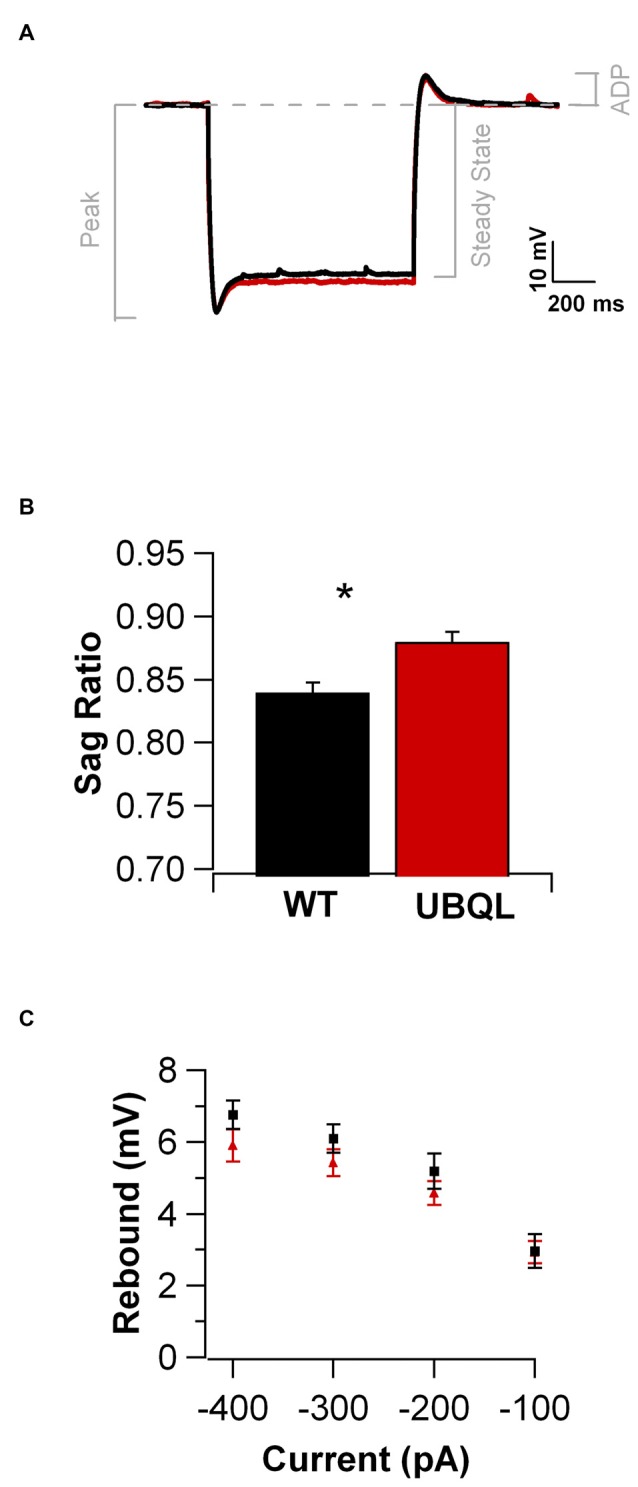
**Membrane responses to hyperpolarizing current injections are similar in UBQLN2^P497H^ and control cells. (A)** Voltage traces in response to negative current injections adjusted to produce the same peak hyperpolarization (−120 mV; for the traces shown here, the injected current was: −450 pA for WT and −250 pA for UBQLN2^P497H^, 1 s) in a cell from a WT (black trace) and one from a UBQLN2^P497H^ mouse (red trace). **(B)** Cells from UBQLN2^P497H^ show a small decrease in the peak to steady state hyperpolarization (the sag ratio was 0.88 ± 0.008 in UBQLN2^P497H^ and 0.84 ± 0.01 in control mice, 14 and 12 cells, respectively, *p = 0.007, Wilcoxon-Mann-Whitney). **(C)** This difference, however did not significantly modify anode break excitability.

**Figure 4:**
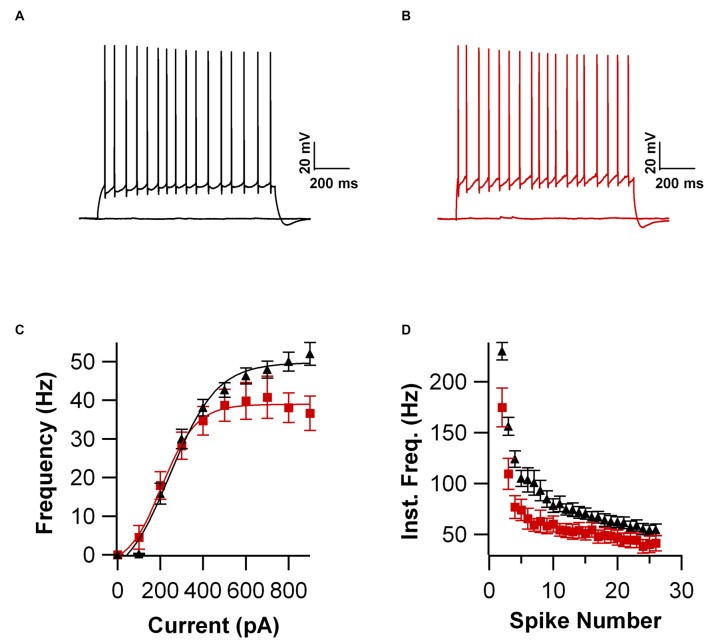
**Firing frequency is reduced in UBQLN2^P497H^ cells. (A,B)** Voltage responses to 1 s long depolarizing current injections (200 pA) in a cell from control mouse **(A)** and one from a UBQL mouse. **(B,C)** Input/output function obtained in 12 cells from WT and 12 from UBQLN2^P497H^ mice. Note the reduction in maximum firing frequency in UBQLN2^P497H^ cells. **(D)** Instantaneous frequency plotted against spike number. For this plot the current injection in each cell was the largest that that did not induce depolarization block. Note the difference both in maximum frequency and in the steady-state values.

**Figure 5:**
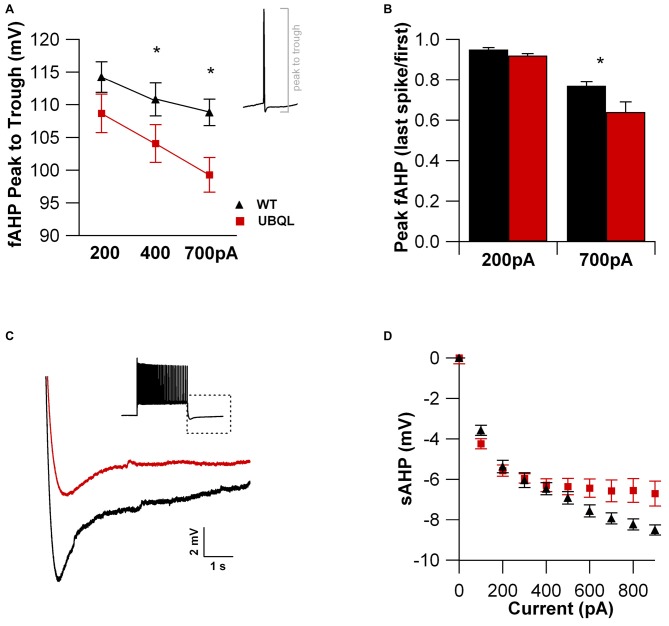
**The fast and slow AHPs are reduced in cells from UBQLN2^P497H^ mice. (A)** The fast afterhyperpolarization (fAHP) amplitude (measured as the voltage change between the peak and trough of the first Action potential (AP), see inset) elicited with 400 pA (*p* = 0.0496, Wilcoxon-Mann-Whitney) or 700 pA (*p* = 0.01, Wilcoxon-Mann-Whitney) current injections was consistently smaller in UBQL recordings. **(B)** When comparing the first and last spike within an AP train, the fAHP reduction was considerably larger in UBQLN2^P497H^ mice, further impairing the ability of these neurons to fire at high frequencies (*p = 0.03, Wilcoxon-Mann-Whitney). **(C)** The slow AHP recorded at the end of an AP train (inset; 700 pA current injection) was markedly reduced in UBQLN2^P497H^ mice for any current injection ≥800 pA, as summarized in panel **(D)**. Traces in **(C)** were obtained with 700 pA current injections.

Intrinsic properties are as important as the synaptic ones to determine overall neuronal function. Therefore, we also compared the intrinsic excitability in CA1 pyramidal cells of WT and UBQLN2^P497H^ mice. First, we investigated the basic membrane properties of these cells. No difference was detected in the resting membrane potential (−63.8 ± 0.7 mV and −63.3 ± 1.0 mV, *n* = 12, 11), capacitance (184.6 ± 7.7 pF and 186.5 ± 17.1 pF *n* = 12, 13), or input resistance (71.7 ± 3.5 MΩ and 70.2 ± 8.2 MΩ in 12 WT and 13 UBQLN2^P497H^ cells respectively; Figure 2). Next, we measured the ratio of the peak to steady-state response to an injection of 1 s long hyperpolarizing current steps (Russo et al., 2007) to estimate the size of the hyperpolarization-activated cationic current (Ih). Both genotypes showed only modest voltage sag in response to hyperpolarizing pulses; the sag ratio, however, was slightly but consistently decreased in UBQLN2^P497H^ mice (Figure 3), suggesting that Ih density was moderately decreased in cells from these mice, although this did not significantly affect the anode break depolarization (Figure 3C). We then measured the firing phenotype of these neurons. Cells were recorded in current clamp and their resting membrane potential was set at –70 mV with injection of negative bias current, when necessary. The input/output function was studied by measuring the voltage responses to injection of square depolarizing current pulses (1 s; 100 pA steps; Figures 4A,B). We found that the maximum firing frequency elicited by saturating current injections was significantly lower in pyramidal cells of UBQLN2^P497H^ mice compared to control cells (Figure 4C), The instantaneous frequency was also reduced and declined significantly faster with each spike in UBQLN2^P497H^ compared with control cells (Figure 4D). In keeping with this finding, the fast afterhyperpolarization (fAHP) that follows the first AP in a train was also significantly reduced in UBQLN2^P497H^ cells over a range of different current injections (in response 400 pA current injections the peak-to-trough voltage change was 104.06 ± 2.9 mV vs. 110.80 ± 2.5 mV in mutant and wild type control animals, respectively (12 cells in each group; *p* = 0.0496); with 700 pA current injections the peak-to-trough voltage change was 99.28 ± 2.7 mV vs. 108.81 ± 2.0 mV in control, 12 and 11 cells, respectively, *p* = 0.01, Figure 5A). Moreover, the amplitude of the fAHP in cells from UBQLN2^P497H^ mice decayed faster within an AP train (the ratio of the first to the last fAHP with 700 pA current injections was 0.64 ± 0.05 and 0.77 ± 0.02, in UBQLN2^P497H^ and WT *n* = 12 and 11, respectively; *p* = 0.03, Figure 5B). The slow afterhyperpolarization (sAHP) at the end of a long spike train was also significantly reduced in cells from UBQLN2^P497H^ mice, but only following current injections ≥800 pA (Figures 5C,D), a level comparable with those at which differences in firing frequency become apparent (see Figure 5C). This suggests that the differences in sAHP may be attributed by and large to the different numbers of spikes within each train rather than to differences in channel density or gating.

**Figure 6:**
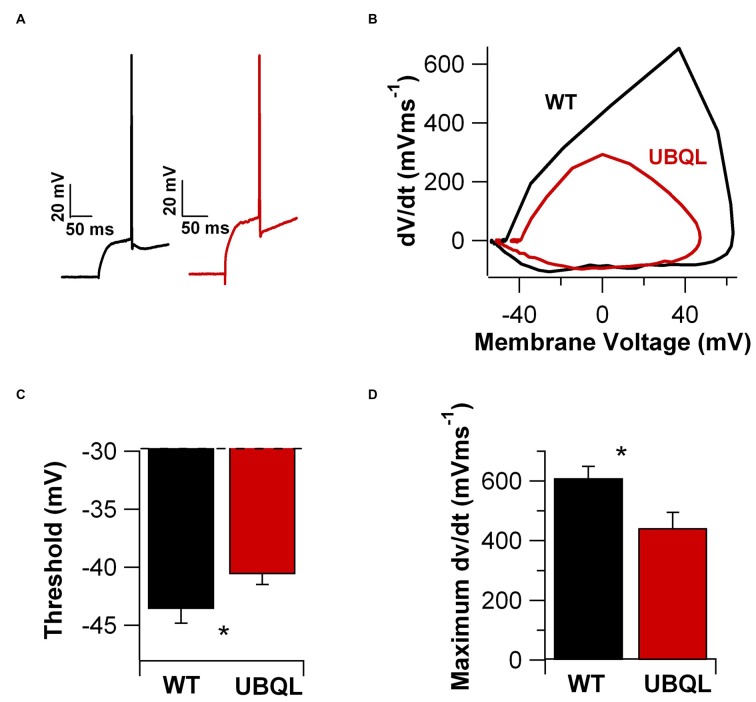
**AP upstroke is blunted in UBQLN2^P497H^ cells. (A)** APs recorded in cells from control (black trace) and UBQLN2^P497H^ (red traces) mice. Resting potential was −70 mV, 200 pA current injections. **(B)** Phase plots of the APs in **(A)**. Note the depolarized threshold (dotted lines) and smaller maximum rate of rise in UBQLN2^P497H^ cells. **(C,D)** Summary bar charts of showing the differences in threshold and the maximum rate of rise (*p = 0.03, Wilcoxon-Mann-Whitney) in 12 controls and 14 UBQLN2^P497H^ cells.

Finally, we examined the properties of the AP upstroke. We found that in cells from UBQLN2^P497H^ mice the AP threshold was more depolarized compared to wild type (−40.5 ± 0.87 mV vs. −43.6 ± 1.2 mV, *n* = 14 and 12, respectively, *p* = 0.03, Figures 6A–C). Consistent with this finding, the maximum dV/dt was also significantly smaller in UBQLN2^P497H^ mice (442.29 ± 52.2 V*s^-1^ vs. 610.0 ± 39.2 V*s^-1^, *n* = 14 and 12 respectively, *p* = 0.03, Figure 6D). Thus, intrinsic excitability is decreased in a number of relevant parameters in UBQLN2^P497H^ cells, possibly contributing to the cognitive deficits.

## Discussion

### Excitability of CA1 Pyramidal Cells in ALS/FTD

Despite the fact that cognitive impairment is relatively frequent in ALS (Ferrari et al., 2011; Lomen-Hoerth, 2011) and frontotemproral dementia is not uncommon, very few studies have investigated the electrophysiological properties of cortical and hippocampal neurons in ALS models. No data are available concerning the properties of hippocampal neurons in any ALS model. A recent article (Saba et al., 2016) reports that intrinsic and synaptic excitability is enhanced in cortical motor neurons from 1 month old mice carrying the G93A substitution in the SOD1 gene and suggests that these findings may mediate the upper motor neuron hyperexcitability in ALS. Using the same mouse model, another group had found overexpression of N-type calcium channels (Pieri et al., 2013) in cultured cortical neurons, which could be suggested as a mechanism of calcium-toxicity mediated cell death, as well as, the increase in intrinsic excitability. The mechanistic significance of these findings, however, is unclear because SOD1-type ALS is primarily a lower motor neuron disease and thus whether these functional alterations may have a causal role is unclear. Indeed, while about 25% of all ALS patients show signs of FTD, patients carrying the SOD1 mutation (the first identified genetic cause of ALS; Deng et al., 1993; Rosen et al., 1993) are less likely to show cognitive signs (Wicks et al., 2009). In this study, however, we used a recently developed mouse model of ALS/FTD (Gorrie et al., 2014) based on a mutation found in human patients (Deng et al., 2011). About 25% of ALS patients affected by this mutation show FTD signs and in some patients the cognitive symptoms are the first to appear (Deng et al., 2011). Accordingly, the transgenic mice carrying the mutation show cognitive symptoms, as well as, altered hippocampal morphology and function (Gorrie et al., 2014). Here we show that synaptic and intrinsic excitability are both decreased in CA1 pyramidal neurons from 1 month old UBQLN2^P497H^ mice. It is not necessarily surprising that two ALS models show opposite electrophysiological changes in pyramidal neurons because, as noted, neither the specific cell types (hippocampal pyramidal cells vs. cortical motor neurons), nor the ALS model (UBQLN2^P497H^ vs. G93A) investigated in this article are the same as those in the previous articles and the SOD1 G93A is a model of a lower motor neuron disease while the UBQLN2^P497H^ is a model of ALS/FTD. On the other hand, our findings of impaired glutamatergic transmission in the hippocampus of P497H mice may represent a mechanism for the hippocampal related cognitive deficits in this model. Indeed, it may be that the functional impairments of hippocampal neurons are the only ones detectable at this young age. Future studies in other CNS areas will be needed to address this point.

### Potential Mechanisms of the Decreased Intrinsic Excitability in UBQLN2^P497H^ Pyramidal Cells

Beside a large reduction of the glutamatergic currents, pyramidal cells from UBQLN2^P497H^ animals also display impairment in intrinsic excitability, as evidenced by their reduced maximum firing frequency, increased AP threshold and accommodation and decreased AHP. A difference in maximum firing frequency is likely the consequence of decreased potassium current density, because potassium channels regulate maximum firing in hippocampal pyramidal cells (Martina et al., 1998; Lien and Jonas, 2003). In CA1 pyramidal cells, the total potassium current is composed of voltage-gated channels, calcium-dependent channels and leakage channels (Storm, 1988; Martina et al., 1998; Taverna et al., 2005). The voltage-gated current has three main functional components: an IA current, largely mediated by KV4 channels, a fast delayed rectifier mediated by Kv3 channels and a slow delayed rectifier mediated by Kv1 and Kv2 channels (Storm, 1988; Martina et al., 1998; reviewed in Martina, 2010). Because AP repolarization is mostly mediated by Kv3-like currents (Mitterdorfer and Bean, 2002), the reduced magnitude of the fAHP that follows the APs suggests that the density of these channels may be reduced in these mice. Indeed, a reduction in the fAHP is already detectable using relatively small current injections (400 pA) that are below the amplitude resulting in significant differences in firing frequency (see Figures 5A,D). On the other hand, the slow hyperpolarization that follows a spike train (sAHP), which in CA1 pyramidal cells is largely mediated by KCa3.1 subunits (King et al., 2015), is also significantly reduced in the UBQLN2^P497H^ mice, but this reduction appears to be largely the consequence of the reduced maximum firing frequency and, consequently, the reduced number of spikes within each prolonged AP train in ALS mice, as it only appears when significant differences in firing frequency are detectable. Thus, the downregulation of this current in UBQLN2^P497H^ mice may not have a causal role in the electrophysiological phenotype. The third major class of potassium channels in CA1 pyramidal cells is composed of the leakage K^+^ conductances, which are important in setting resting membrane potential and input resistance (Taverna et al., 2005). Because these functional parameters are unchanged in ALS mice, it is likely that the leakage channels are not affected. Beside potassium currents, our data suggest that Ih, the hyperpolarization activated excitatory current (Maccaferri et al., 1993) may also be impaired in these mice. Although minor, a decrease in Ih would still contribute to the reduced ability of cells to fire when released from inhibition. Finally, the more depolarized AP threshold and the smaller value of the maximum dv/dt suggest that the sodium current is either smaller or displays altered kinetics. Therefore, the reduction in intrinsic excitability is detectable in several functional states of ALS neurons and is likely due to changes in all major voltage-gated conductances.

### Do the Electrophysiological Changes have a Causal Role in the Progression of the Disease?

We found that both the synaptic and the intrinsic excitability are decreased in CA1 pyramidal cells in 1 month old UBQLN2^P497H^ mice. How the mutation may lead to these specific impairments is not clear, but it can be suggested that the observed impairment of the synaptic proteasome system may affect the timely turnover of membrane channels and receptors. Because the pathology associated with the mutation appears to eminently affect dendritic spines, the synaptic alterations are not surprising. The decreased intrinsic excitability, however, may be more surprising. In fact, while changes in synaptic excitability often lead to modulation of intrinsic excitability, this is usually in the form of homeostatic plasticity (Turrigiano, 1999; Ren et al., 2016). Here, however, this is not the case as the changes in intrinsic and synaptic excitability show the same polarity (reduced excitability). Alterations in firing pattern and AP shape have implications that go beyond electrical signaling. CA1 pyramidal cells are endowed with a large number of voltage-gated calcium channels (Christie et al., 1995; Magee and Johnston, 1995; Metz et al., 2005; Radzicki et al., 2013). Thus, altered excitability—due to either synaptic or intrinsic changes—may lead to important changes in the calcium homeostasis. Impaired calcium regulation may then modulate ion channel gene expression (Cohen and Greenberg, 2008), thus transforming a potentially reversible alteration in excitability into a self-sustaining functional dysregulation; this is also supported by the report that depolarization of cultured cortical neurons affects the expression of DNA methyltransferases (Sharma et al., 2008), which control DNA methylation and, thus, gene expression. This hypothesis is particularly important in the context of a possible therapeutic approach because these electrophysiological changes are observed in 1 month old animals when no clear cellular pathology is observed (Gorrie et al., 2014). Thus, if the calcium-dependent (dys)regulation of gene expression was indeed taking place in these neurons, it may be suggested that by controlling the neuronal excitability, which regulates calcium entry, or by directly regulating the calcium release from intracellular calcium stores it could be possible to stave off the subsequent gene expression dysregulation and potentially even prevent, or at least slow down, the progression of the disease.

## Author Contributions

DR: electrophysiological recordings and data analysis; drafting the manuscript. EL: creating the mouse model. H-XD: creating the mouse model; critical discussions of the project. TS: general development of the project; providing financial support; drafting the manuscript. MM: general development of the project; financial support; experimental design, writing the manuscript.

## Funding

This work was supported by NIH grant NS078504 (TS).

## Conflict of Interest Statement

The authors declare that the research was conducted in the absence of any commercial or financial relationships that could be construed as a potential conflict of interest. The reviewer AM and handling Editor declared their shared affiliation, and the handling Editor states that the process nevertheless met the standards of a fair and objective review.
